# Metagenomic 16S rRNA amplicon data of gut microbial diversity in three species of subterranean termites (*Coptotermes gestroi, Globitermes sulphureus* and *Macrotermes gilvus)*

**DOI:** 10.1016/j.dib.2023.108993

**Published:** 2023-02-16

**Authors:** Qurratu'Aini Syasya Shamsuri, Abdul Hafiz Ab Majid

**Affiliations:** a Household and Structural Urban Entomology Laboratory, Vector Control Research Unit, School of Biological Sciences, Universiti Sains Malaysia (USM), Minden, Pulau Pinang 11800, Malaysia; b Centre for Insect Systematics (CIS), Faculty of Science and Technology, Universiti Kebangsaan Malaysia (UKM), Bangi, Selangor 43600, Malaysia

**Keywords:** Coptotermes gestroi, Globitermes sulphureus, Macrotermes gilvus, Subterranean termites, Gut microbial diversity, Metagenomics

## Abstract

In this paper, we present the metagenomic dataset of gut microbial DNA of the lower group of subterranean termites, i.e. *Coptotermes gestroi*, and the higher groups, i.e. *Globitermes sulphureus* and *Macrotermes gilvus*, in Penang, Malaysia. Two replicates of each species were sequenced using Next-Generation Sequencing (Illumina MiSeq) and analysed via QIIME2. The results returned with 210,248 sequences in *C. gestroi*, 224,972 in *G. sulphureus*, and 249,549 in *M. gilvus*. The sequence data were deposited in the NCBI Sequence Read Archive (SRA) under BioProject number of PRJNA896747. The community analysis showed that Bacteroidota is the most abundant phylum in *C. gestroi* and *M. gilvus*, while Spirochaetota is prevalent in *G. sulphureus*.

**Specifications Table**

**Table:** 

Subject	Microbiology: Microbiome
Specific subject area	16S rRNA gene amplicon metagenome sequencing of gut microbial diversity in three species of subterranean termites, i.e. *Coptotermes gestroi, Globitermes sulphureus,* and *Macrotermes gilvus*.
Type of data	Figures, table, and 16S rDNA Illumina sequences.
How the data were acquired	16S rDNA Illumina sequencing followed by community metagenome analysis.
Data format	Raw: FASTQ file.
Description of data collection	The gut microbial DNA was extracted using GeneAll® Exgene™ Stool DNA mini kit (GeneAll Biotechnology, Korea). 16S rDNA metagenomic sequencing was done via the Illumina MiSeq platform.
Data source location	Institution: Universiti Sains Malaysia (USM) City/Town/Region: Penang Country: Malaysia GPS coordinate for collected samples: 5°21′14.5"N 100°18′01.1"E, 5°21′23.1"N 100°17′52.4"E and 5°21′19.1"N 100°17′36.7"E
Data accessibility	Repository name: NCBI SRA Data identification number: PRJNA896747 Direct URL to data: https://www.ncbi.nlm.nih.gov/sra/PRJNA896747

## Values of the Data

• The 16S metagenomics data provide taxonomic data on microbial diversity and abundance in the guts of *Coptotermes gestroi, Globitermes sulphureus,* and *Macrotermes gilvus* collected in urban area.
• Results obtained are particularly useful for urban and agricultural biotechnologist in Malaysia.
• This study offers potential avenues for further research in discovering novel genes that may contribute towards the coding of enzymes or protein involved in nutrients amplification, biomass degradation, and antibiotic resistance.

## 1 Objective

*Coptotermes gestroi* (*C. gestroi*) is a primary pest from the termite family which is commonly found in home premises and urban areas in Malaysia. This species takes approximately ten to thirteen weeks to be controlled using multiple commercial formulations of baiting treatment [6,14]. *Globitermes sulphureus* (*G. sulphureus*) and *Macrotermes gilvus* (*M. gilvus*) also infest home premises and urban areas. However, unlike *C. gestroi*, both of these species generally take an extended period to be controlled using baiting treatment. Hence, it is crucial to characterise the microbial composition inhabiting the guts to understand the bacterial species that might involve in the survivability of bait-treated termites. The purpose of this study is to determine the pre-treatment microbial community of the three species of subterranean termites, namely *C. gestroi, G. sulphureus*, and *M. gilvus*.

## 2 Data Description

**Fig. 1:**
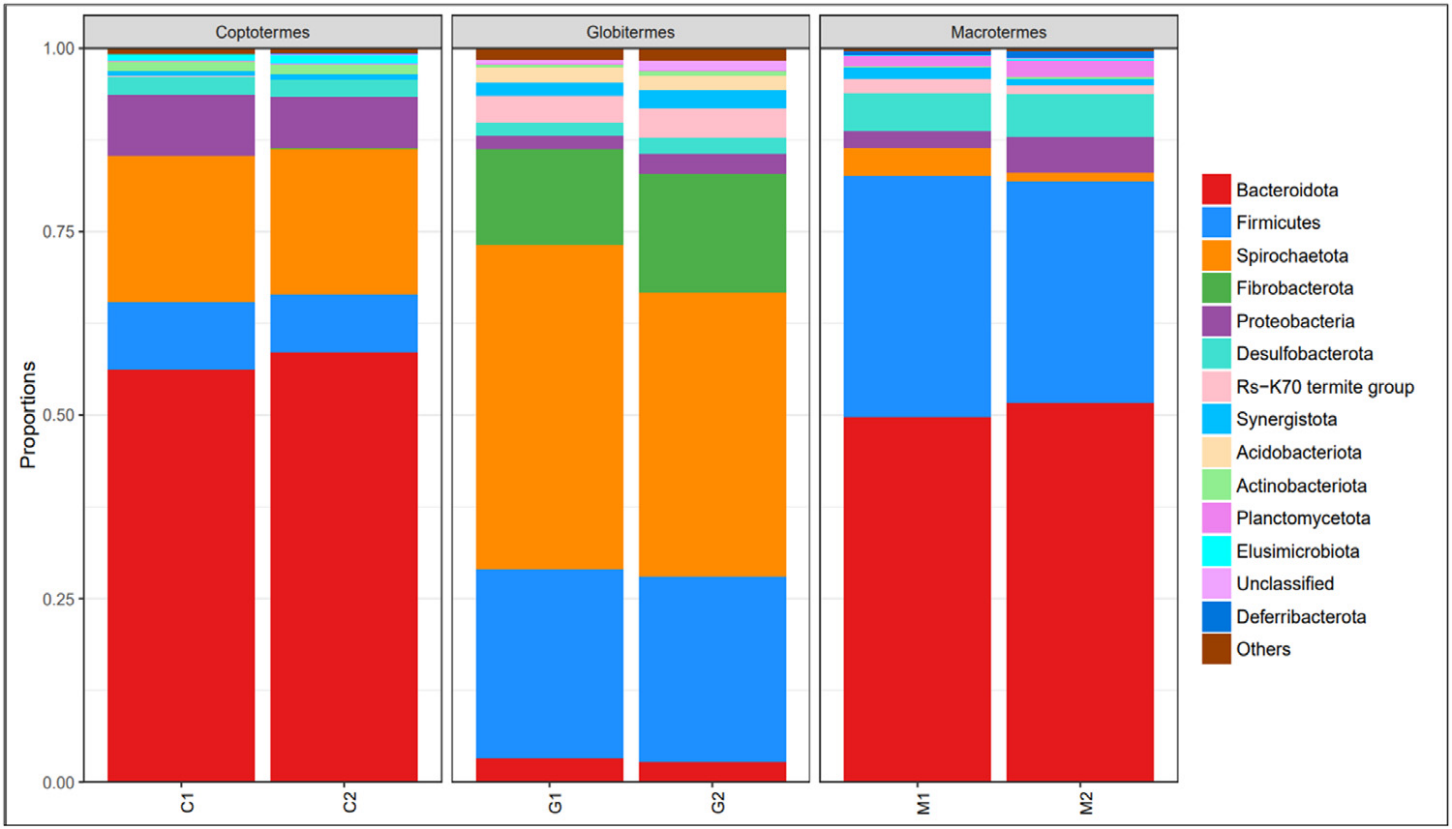
The stacked bar chart shows the taxonomic abundance at phylum level of gut microbes in the three subterranean termites species. X-axis represents two biological replicates of the pooled samples for each species, which are C1 and C2 (*C. gestroi*), G1 and G2 (*G. sulphureus*), and M1 and M2 (*M. gilvus*). Y-axis is the taxon abundance. Phyla with percentage less than 1% were clubbed and assigned in ‘Others’.

The dataset in Table 1 consists of overall sequences and average base pairs obtained through Illumina 16S bacterial metagenomic sequencing (Illumina MiSeq) of the lower group (*C. gestroi*) and the higher groups (*G. sulphureus* and *M. gilvus*) of subterranean termites collected from USM, Penang, Malaysia. The community analysis revealed that Bacteroidota is the most predominant microbial phylum of the gut microbial composition in *C. gestroi* and *M. gilvus*, while Spirochaetota is the most predominant one in *G. sulphureus*. The dataset table and stacked taxon bar chart for other gut phyla are depicted in Table 1 and Figure 1, accordingly.

**Table 1:** The number of sequences, base pairs, and the average length of *C. gestroi* (C1 and C2), *G. sulphureus* (G1 and G2), and *M. gilvus* (M1 and M2).

Sample	Subterranean termite species	Sequences	Bases (bp)	Average length (bp)
C1	*C. gestroi*	109,472	46,254,556	422.52
C2	*C. gestroi*	100,776	42,594,628	422.67
G1	*G. sulphureus*	124,249	52,486,488	422.43
G2	*G. sulphureus*	100,723	42,527,916	422.23
M1	*M. gilvus*	100,187	41,919,559	418.41
M2	*M. gilvus*	149,362	62,513,822	418.54

**Fig. 2:**
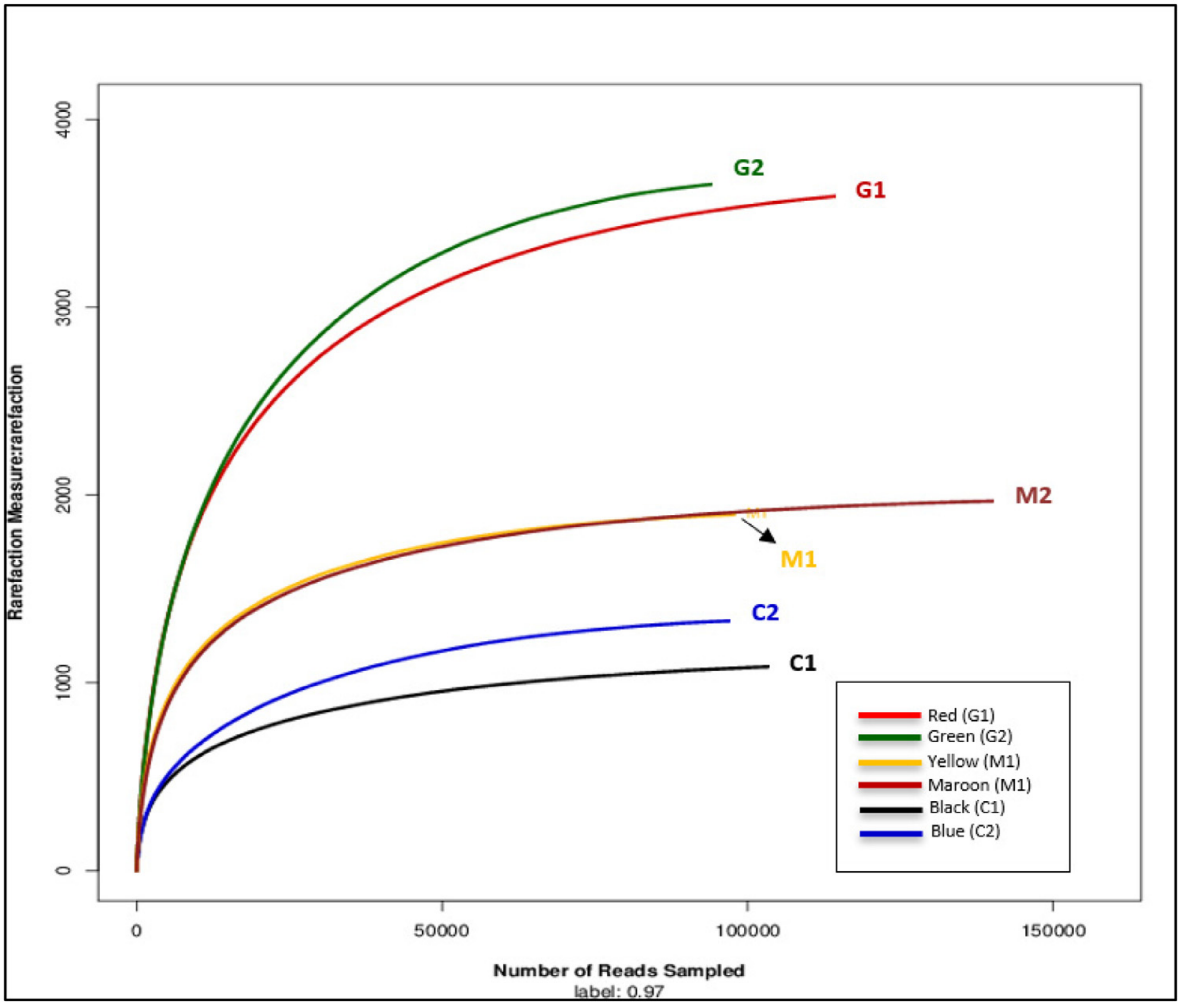
Rarefaction curves of the six pooled samples at 97% sequence similarity illustrate the species richness of gut microbiota in the three subterranean termites species. X-axis represents the number of reads/ samples. Y-axis shows the measures of detected species richness which were calculated with Chao1 index. As can be seen, the M1 curves (yellow) overlapped with M2 curves (maroon). Table 2 provides more details about the labelling of the colour-coded curves and Chao1 index.

Rarefaction allows the calculation and determination of species richness, alpha diversity, independent sequencing depth of the community for each sample. The resulting construction of rarefaction curves would explain the adequacy of the sequencing depth in estimating the species diversity. An ascending curve indicates that a large fraction of the species diversity remains to be discovered, while a flatter curve represents a sufficient sequencing depth which has reached the saturation state [5]. Table 2 shows the species richness data and Figure 2 illustrates the alpha diversity estimators and rarefaction graph.

**Table 2:** Species richness and alpha diversity estimators of the gut microbial communities in the three species of subterranean termites.

Sample	Curve colour	Chao1	Shannon index	Simpson index
C1	Black	1144.1866	6.3602	0.0846
C2	Blue	1350.9009	6.3260	0.0879
G1	Green	3695.5908	9.5249	0.0047
G2	Red	3705.0130	9.4580	0.0054
M1	Black	1907.3626	8.6602	0.0091
M2	Blue	2001.6206	8.4033	0.0127

## 3 Experimental Design, Materials, and Methods

### 3.1 Insect Sampling and DNA Extraction

The three subterranean termite species, namely *C. gestroi, M. gilvus*, and *G. sulphureus*, are common insect pests in urban habitation, thus making them suitable for this research. All samples were collected from the termite underground stations located in USM, Penang, Malaysia, except for those of the *G. sulphureus* which were excavated from the termite mound. These samples were identified based on the standard taxonomic keys of the soldier caste due to the distinctive characteristics among the genera [1,[9], [10], [11]]. Approximately 50 individuals of termite workers per species were cleaned twice with ethanol and once with sterile distilled water to discard any soil debris and to minimise the risk of contamination by the soil microbiomes. Next, the guts were carefully extracted from the abdomens of 20 workers using sterilised forceps and pooled together as a biological replicate in which two replicates per species were needed in this study [8]. Prior to the homogenisation, the samples were kept for 15 minutes below −20°C, and then the genomic DNA was extracted according to the manufacturer's protocols using the GeneAll® Exgene™ Stool DNA mini kit (GeneAll Biotechnology, Korea) [15].

### 3.2 PCR Amplification and Illumina Sequencing

The extracted gDNA from the six pooled samples were sent to Nuclix Biosolution (located in Melaka, Malaysia) for Illumina sequencing. The 16S V3-V4 marker bacteria region was PCR amplified at the following temperature setting: 95°C for two minutes, 25 cycles at 95°C for 30 seconds, 55°C for 30 seconds, 72°C for 30 seconds, and a final extension at 72°C for five minutes. The prepared libraries were pooled in equimolar and paired-end sequenced (2 × 250/300 bp) on the MiSeq platform following the standard protocols. Next, the raw FASTQ files were analysed in QIIME2 (version 2022.8.3) [3]. Reads which received an average quality score of less than 20 were trimmed using Trimmomatic software with an alternative sliding window whereby those shorter than 50bp were discarded [2]. Short and paired-end reads were merged into longer reads using Fast Length Adjustment of Short Reads, i.e. FLASH [12]. Next, the overlapped sequences with more than 10 bp in length were assembled and the unassembled reads were subsequently removed. Due to the numerous reads processed, the Operational Taxonomic Unit (OTU) clustered the data sets according to the 97% similarity cut-off using Usearch [7]. Then, the RDP classifier was used to analyse the taxonomy of the 16s rRNA gene sequences against the Silva (SSU123) 16s rRNA database using the 0.7 confidence threshold. The species richness and alpha diversity covered in each sample were estimated using Chao1, Shannon index, and Simpson index using Mothur [13], as shown in Table 2. The rarefaction curves of the six pooled samples (Figure 2) were constructed with R software [4].

## Ethics Statements

Our work does not involve studies with animals and humans.

## CRediT authorship contribution statement

**Qurratu'Aini Syasya Shamsuri:** Methodology, Investigation, Data curation, Formal analysis, Writing – original draft, Writing – review & editing. **Abdul Hafiz Ab Majid:** Conceptualization, Supervision, Project administration, Resources, Funding acquisition, Writing – review & editing.

## Declaration of Competing Interest

The authors declare that they have no known competing financial interests or personal relationships that could have appeared to influence the work reported in this paper.

## Data Availability

Gut microbial diversity in the lower group of subterranean termites, Coptotermes gestroi and the higher group, Globitermes sulphureus and Macrotermes gilvus (Original data) (NCBI)
